# Rejection after BKPyV DNAemia—Are We Treating Too Cautiously?

**DOI:** 10.3389/ti.2025.15122

**Published:** 2025-09-18

**Authors:** Wouter T. Moest, Aiko P. J. de Vries, Aline L. van Rijn, Danny van der Helm, Jesper Kers, Mariet C. W. Feltkamp, Joris I. Rotmans

**Affiliations:** ^1^ Department of Internal Medicine, Leiden University Medical Center, Leiden, Netherlands; ^2^ Transplant Center, Leiden University Medical Center, Leiden, Netherlands; ^3^ Department of Medical Microbiology and Infection Prevention, Leiden University Medical Center, Leiden, Netherlands; ^4^ Department of Pathology, Leiden University Medical Center, Leiden, Netherlands; ^5^ Department of Pathology, Amsterdam UMC, University of Amsterdam, Amsterdam, Netherlands

**Keywords:** kidney transplantation, biopsy proven acute rejection, BK polyomavirus, rejection treatment, graft function

Dear Editors,

After kidney transplantation, BK polyomavirus (BKPyV) DNAemia affects approximately 10%–30% of recipients. In a subset of these cases (1%–10%), BKPyV DNAemia progresses to BK polyomavirus-associated nephropathy (BKPyVAN), marked by renal impairment and the risk of graft failure [1–4]. Treatment primarily involves the reduction of immunosuppression since effective antiviral therapy against BKPyV is lacking [5–8].

However, a clinical dilemma arises when patients develop biopsy-proven acute rejection (BPAR) following an episode of BKPyV DNAemia. In our experience, clinicians are often hesitant to initiate full immunosuppressive therapy due to concerns about reactivating BKPyV infection. This creates uncertainty about whether a more restrained approach to rejection treatment is warranted in this setting.

To explore this, we conducted a single-center, retrospective cohort study including adult kidney transplant recipients (KTRs) transplanted at the Leiden University Medical Center (LUMC) between 2011 and 2020. Patients with primary non-function, multi-organ transplants, missing follow-up data, or participation in investigational immunosuppressive trials were excluded.

Patients were categorized into three groups [1]: those with biopsy-proven acute rejection (BPAR) preceded by BKPyV DNAemia (BKPyV-BPAR) [2], those with BPAR occurring ≥6weeks post-transplantation without prior BKPyV DNAemia (BPAR-only), and [3] those without BPAR during follow-up (No-BPAR). The ≥6-week threshold for the BPAR-only group was chosen because BKPyV DNAemia is typically not present before this period. Consequently, rejection episodes occurring from week 6 are more likely to be pathophysiological and clinically comparable to those preceded by BKPyV DNAemia.

Graft outcomes, including eGFR over time and graft loss at 5 years, were compared between groups. To assess rejection management, we evaluated adherence to our center’s treatment protocol. Therapy was considered “less than protocol” if prescribed doses were reduced or agents were withheld entirely. In addition, to gain insight into potential delays in treatment of rejection, we calculated the time interval between a ≥20% rise in serum creatinine and the initiation of rejection treatment.

Data were extracted from the transplantation database, which is directly linked to structured data of electronic health record and pathology department. Data were analyzed using Chi-square test, One-way ANOVA, and linear Mixed-Effects Model (LMM) to assess longitudinal eGFR trends at 6 weeks, 6 months, 1 year, 3 years and 5 years after transplantation.

At our center, BKPyV DNAemia is screened at 1.5, 3, 6, and 12 months after kidney transplantation using quantitative real-time PCR on serum samples. If BKPyV DNAemia is detected, monitoring frequency increases to every 2 weeks. For viral loads <10E4 copies/mL, calcineurin inhibitor (CNI) trough levels are checked and, if on target, prednisone is tapered to 5 mg/day and mycophenolate mofetil (MMF) dose is halved. For loads >10E4 copies/mL, the CNI dose is halved and MMF is discontinued. After two consecutive undetectable serum BKPyV loads, immunosuppression is increased to standard protocol trough levels.

BPAR was defined by histopathological assessment according to the Banff classification of the time of biopsy. In cases with suspected BKPyVAN—typically BKPyV DNAemia >4 log copies/mL with rising creatinine—a kidney biopsy was performed. The distinction between TCMR Banff 1A and BKPyVAN can be challenging, as both can present with interstitial inflammation and tubulitis. To reduce misclassification, SV40-staining was systematically applied in biopsies with tubulointerstitial inflammation. A positive SV40-staining in combination with detectable BKPyV DNAemia is considered as BKPyVAN. Whereas negative SV40-staining in combination of undetectable BKPyV DNAemia in the setting of tubilitis favors TCMR.

This study was reviewed by the LUMC ethics committee, which concluded that formal approval was not required (ID:131830).

A total of 968 KTRs were included in this study. Of these, 870 (89.9%) were classified as No-BPAR, 66 (6.8%) as BPAR-only, and 32 (3.3%) as BKPyV-BPAR. Patients in both rejection groups (BPAR-only and BK-BPAR) were younger, more frequently re-transplanted, and more often immunized (PRA >5%) compared to those in the No-BPAR group. When examining rejection within the groups, mixed rejection was more common in the BKPyV-BPAR group compared to the BPAR-only group (31.3% vs. 10.6%), whereas T-cell mediated rejection (TCMR) was more common in the BPAR-only group (74.2% vs. 59.4%), p = 0.038. In the BKPyV-BPAR group, BPAR occurred on average 210 ± 429 days after BKPyV DNAemia.

With regard to rejection treatment, patients in the BKPyV-BPAR group more often received less immunosuppressive treatment than recommended by the local rejection management protocol (details in Supplementary Table S1a), compared to those in the BPAR-only group (19.7% vs. 56.3%, p < 0.001). Moreover, the initiation of rejection treatment — measured from the time of a ≥20% rise in serum creatinine — was significantly delayed in the BKPyV-BPAR group compared to the BPAR-only group (average of 16.5 ± 23.1 vs. 7.8 ± 10.5 days, p = 0.012). Supplementary Table S1b further details how maintenance immunosuppression was adjusted before and after rejection in the BKPyV-BPAR group. When further stratifying the BKPyV-BPAR group based on the timing of rejection treatment, patients who received therapy within ≤7 days after the 20% increase in serum creatinine showed a mean eGFR improvement of 6.6 ± 6.7 mL/min/1.73 m^2^ at day 7 after rejection treatment. In contrast, in patients where treatment was initiated >7 days after the creatinine rise, the mean eGFR increase was only 0.4 ± 7.6 mL/min/1.73 m^2^ (p = 0.03).

When analyzing graft function, graft loss at 5-year occurred in 3.9% of No-BPAR patients, 27.7% in the BPAR-only group, and 45.0% in the BKPyV-BPAR group (p < 0.001). In univariate LMM analysis, patients in the BKPyV-BPAR group showed a non-significant trend towards lower eGFR compared to the BPAR-only group (−1.6 mL/min; 95%CI: −8.8 to 5.6; p = 0.659), which was similar in multivariate analysis (−1.0 mL/min; 95%CI: −7.3 to 5.3; p = 0.750). The eGFR trajectories over time for the three groups are displayed and illustrated in Supplementary Table S2; Figure 1.

**FIGURE 1:**
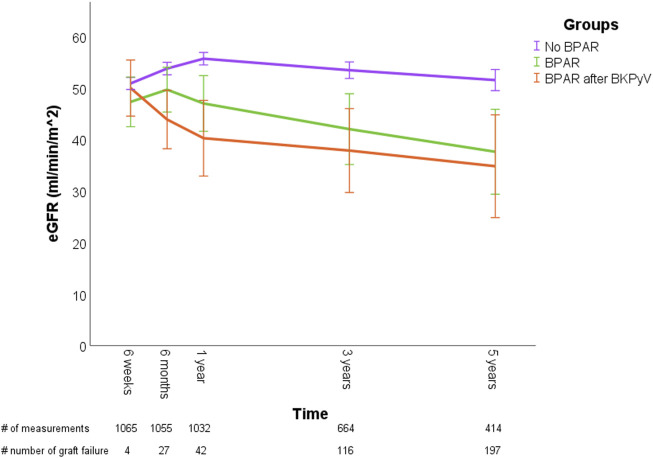
Graft outcomes in kidney transplant patients without BPAR, with BPAR, and BPAR preceded by BKPyV DNAemia. Graphs shows the mean eGFR (ml/min/1.73 m^2) at 6 weeks, 6 months, 1, 3, and 5 years after transplantation. Error bars display 95% confidence interval for the mean. The number of measurements at each time point is shown below the x-axis, additionally, the number of graft failures, defined as return to dialysis or death is indicated separately.

When examining the cause of graft loss, rejection was the predominant reason (91.7%) in the BKPyV-BPAR group. Importantly, no cases of graft loss were attributed to BKPyVAN (Supplementary Table S2).

In addition, the occurrence of BKPyV DNAemia after rejection therapy was examined in both groups. In the BPAR group, 4 patients (6.2%) experienced a recurrence of BKPyV DNAemia following rejection treatment, including 1 patient who developed BKPyVAN. The average peak viral load was 4.1 ± 1.9 log copies/mL, with an average duration of 278 ± 86 days. Recurrence occurred on average 193 ± 130 days after initiation of rejection therapy. In the BPAR preceded by BKPyV DNAemia group, 3 patients (9.7%) developed recurrent BKPyV DNAemia, with an average peak load of 2.8 ± 0.6 log copies/mL and a mean duration of 90 ± 22 days. Recurrence was observed on average 240 ± 380 days after rejection treatment.

An explanation for the limited recurrence of BKPyV DNAemia may be that primary BKPyV episodes do not merely reflect a state of over-immunosuppression, but rather a first-time infection with a novel, previously unencountered BKPyV subtype. Once this initial infection has resolved, protective immunity against that specific strain may limit the risk of recurrence. Supporting this hypothesis, Schmitt et al. demonstrated that VP1 gene sequences in urine samples from 20 matched donor-recipient pairs were completely identical after transplantation, underscoring the role of donor-derived BKPyV and the possible transmission of a new subtype to the recipient [9].

Our study has some limitations, including its retrospective design, a relatively small subgroup size, and limited BKPyV screening up to 1 year post-transplant, potentially missing later DNAemia. In most cases, the cause of graft failure was confirmed by recent biopsy or nephrectomy findings; however, in a few cases it was inferred from older biopsies (>2 months prior). As BKPyV DNAemia was largely absent in the interim, graft loss due to BKPyVAN appears unlikely.

In conclusion, this study shows that patients in whom BPAR was preceded by BKPyV DNAemia experienced both delayed initiation and less intensive rejection treatment, compared to patients with BPAR without prior BKPyV DNAemia. Despite a higher rate of graft loss in this group, the majority of which was attributed to rejection, while no grafts were lost due to BKPyVAN. These findings suggest that conservative rejection management in the context of prior BKPyV needs to be re-evaluated. Further prospective studies are needed to define the optimal management of rejection in the setting of prior BKPyV DNAemia and to better understand the interplay between BKPyV and alloimmunity.

## Data Availability

The raw data supporting the conclusions of this article will be made available by the authors, without undue reservation.
