# Supplementary material for: Rejection after BKPyV DNAemia—Are We Treating Too Cautiously?
Source: Transpl Int. 2025 Sep 18;38:15122. doi: 10.3389/ti.2025.15122 (PMC12488524; doi:10.3389/ti.2025.15122)
Supplement: Supplementary file 1 [file Table1.docx]

Table 1a. Comparison of Rejection Management between patients with BPAR and BPAR preceded by BKPyV DNAemia

|  | BPAR  (n = 66) | BPAR preceded by BKPyV DNAemia  (n = 32) | p-value |
| --- | --- | --- | --- |
| Rejection Treatment (n, %) |  |  | **<0.001*** |
| Below standard | 12 (18.2%) | 18 (56.3%) |  |
| According to standard | 50 (75.8%) | 14 (43.8%) |  |
| Above standard | 4 (6.1%) | 0 (0%) |  |
|  |  |  |  |
| Below Standard Treatment Specified (n,%) |  |  |  |
| No MPS | 4 (33.3%) | 7 (38.9%) |  |
| No ALM | 7 (58.3%) | 8 (44.4%) |  |
| Reduced MPS^1^ | 1 (8.3%) | 1 (5.6%) |  |
| Reduced ALM^2^ | 0 (0%) | 2 (11.1%) |  |

P-values were calculated using *the chi-square test. P-values < 0.05 were considered statistically significant. (1) Reduced MPS consisted of, 3times a day 500mg instead of 3 times a day 1000mg, (2) Reduced ALM consisted of one time 15mg instead of 30mg. Abbreviations: BPAR, biopsy-proven acute rejection; BKPyV, BK polyomavirus; n, number; SD, standard deviation; MPS, methylprednisone; ALM, alemtuzumab

Tabel 1b. Changes in maintenance immunosuppression before and after rejection following BKPyV DNAemia

| Before/After | Pred/MMF/Tac | Pred/MMF/Csa | | Pred/EVL/Tac | | | Pred/EVL/Csa | Pred/MMF/Bela | Pred/Tac | Pred/Csa | Pred/MMF | Total maintenance after rejection |
| --- | --- | --- | --- | --- | --- | --- | --- | --- | --- | --- | --- | --- |
| Pred/MMF/Tac | 4 (12.5%) | | 1 (3.2%) | | 1 (3.2%) |  | |  | 1 (3.2%) |  |  | 7 (22.9%) |
| Pred/MMF/Csa | 2 (6.3%) | | 7 (22.9%) | |  |  | |  |  |  | 1 (3.2%) | 10(31.3%) |
| Pred/MMF/Bela |  | | 1 (3.2%) | |  |  | | 1 (3.2%) |  |  |  | 2 (6.3%) |
| Pred/EVL/Tac |  | |  | | 1 (3.2%) |  | |  |  |  |  | 1 (3.2%) |
| Pred/Aza/Tac |  | |  | |  |  | |  | 1 (3.2%) |  |  | 1 (3.2%) |
| Pred/Tac | 2 (6.3%) | |  | | 3 (9.4%) |  | |  |  |  |  | 5 (15.6%) |
| Pred/Csa |  | | 1 (3.2%) | |  | 1 (3.2%) | |  |  | 1 (3.2%) |  | 3 (9.4%) |
| MMF/Tac | 1 (3.2%) | |  | |  |  | |  |  |  |  | 1 (3.2%) |
| EVL/Tac |  | |  | |  |  | |  | 1 (3.2%) |  |  | 1 (3.2%) |
| Pred |  | |  | |  |  | |  | 1 (3.2%) |  |  | 1 (3.2%) |
| Total maintenance before rejection | 9 (28.1%) | | 10(31.3%) | | 5 (15.6%) | 1 (3.2%) | | 1 (3.2%) | 4 (12.5%) | 1 (3.2%) | 1 (3.2%) |  |

Table 1b. Values are presented as n (%). Abbreviations: Pred. Prednisone, MMF. Mycophenol Motefill, TAC. Tacrolimus, EVL. Everolimus, CsA. Ciclosporine A, Aza. Azathioprine, Bela. Belatacept,

|  |  | No BPAR  (N=870) | BPAR  (n = 66) | BPAR preceded by BKPyV DNAemia  (n = 32) | | p-value | | Post-HOC p-value |
| --- | --- | --- | --- | --- | --- | --- | --- | --- |
|  |  |  |  |  | |  | |  |
| 6 Weeks | eGFR | 50.9 (17.5) | 47.3 (19.4) | 50.0 (15.1) | | 0.281** | |  |
|  | proteinuria | 0.3 (0.4) | 0.5 (0.6) | 0.3 (0.3) | | **0.049**** | | 0.127 |
| 6 Months | eGFR | 53.7 (18.2) | 49.7 (17.3) | 43.9 (15.5) | |  | |  |
|  | proteinuria | 0.3 (0.5) | 0.4 (0.4) | 0.3 (0.3) | |  | |  |
| 1 Year | eGFR | 55.7 (17.9) | 47.0 (21.3) | 40.2 (19.7) | |  | |  |
|  | proteinuria | 0.3 (0.4) | 0.4 (0.5) | 0.7 (1.1) | |  | |  |
| 3 Years | eGFR | 53.4 (18.9) | 42.0 (22.0) | 37.8 (18.3) | |  | |  |
|  | proteinuria | 0.3 (0.5) | 0.8 (1.3) | 1.1 (2.2) | |  | |  |
| 5 Years | eGFR | 51.5 (19.0) | 37.6 (20.2) | 34.8 (17.0) | |  | |  |
|  | proteinuria | 0.4 (0.6) | 0.8 (1.7) | 0.7 (0.5) | |  | |  |
|  |  |  |  |  | |  | |  |
| LMM univariate | | +5.0 (0.8 to 9.3) p=**0.020**^ Reference -2.2 (-9.3 to 5.0) p=0.552^ | | | | |  | |
| LMM multivariate | | +6.6 (2.9 to 10.4) p<**0.001**^^ Reference -1.3 (-7.5 to 4.9) p=0.680^^ | | | | | | |
| 5-Year mortality | | 62 (12.6%) | 5 (10.6%) |  | 0 (0%) | | 0.225* | |
| 5-Year graft failure | | 19 (3.9%) | 13 (27.7%) |  | 9 (45.0%) | | **<0.001*** 0.167 | |
|  | |  |  |  |  | |  | |
| Resasons for graft failure | |  |  |  |  | | **<0.001*** 0.254 | |
| RejecTion | | 0 (0%) | 12 (75.0%) |  | 11 (91.7%) | |  | |
| BKPyVAN | | 2 (7.7%) | 0 (0%) |  | 0 (0%) | |  | |
| Other | | 24 (92.3%) | 4 (25.0%) |  | 1 (8.3%) | |  | |

Table 2. Graft outcomes and mortality in kidney transplant patients without rejection, with biopsy-proven rejection, and with biopsy-proven rejection preceded by BKPyV DNAemia.

Data are shown as mean (SD) or n (%). P-values are calculated with *Chi-square test and **One-way ANOVA, In the event of statistical significance, a post-hoc test was performed, comparing: (a) BPAR with BPAR preceded by BKPyV DNAemia. P-values < 0.05 were considered statistically significant. Linear mixed model analysis (LMM) to compare average eGFR (ml/min/m^2^) over time (6 weeks, 6 months, 1 year, 3 years and 5 years after transplantation), comparing patients with BPAR to patients without BPAR, and patient with BPAR preceded by BKPyV DNAemia. ^Fixed effects were used for the different patient groups and time. ^^Fixed effects were used for age for both recipient and donor, donor gender, delayed graft function (DGF), time, different patient groups and the interaction between time and recipient age, and between different patient groups and time, Patients were designated as a random effect. Proteinuria is in grams collected in 24-hour urine. Graft failure was defined as requiring renal replacement therapy and/or retransplantation and not caused by death.
